# Supplementary material for: Microvascular pathology in the spinal cord of severe spinal muscular atrophy patients
Source: Acta Neuropathol Commun. 2026 Feb 15;14:65. doi: 10.1186/s40478-026-02232-y (PMC13011495; doi:10.1186/s40478-026-02232-y)
Supplement: Supplementary file 1 [file 40478_2026_2232_MOESM1_ESM.docx]

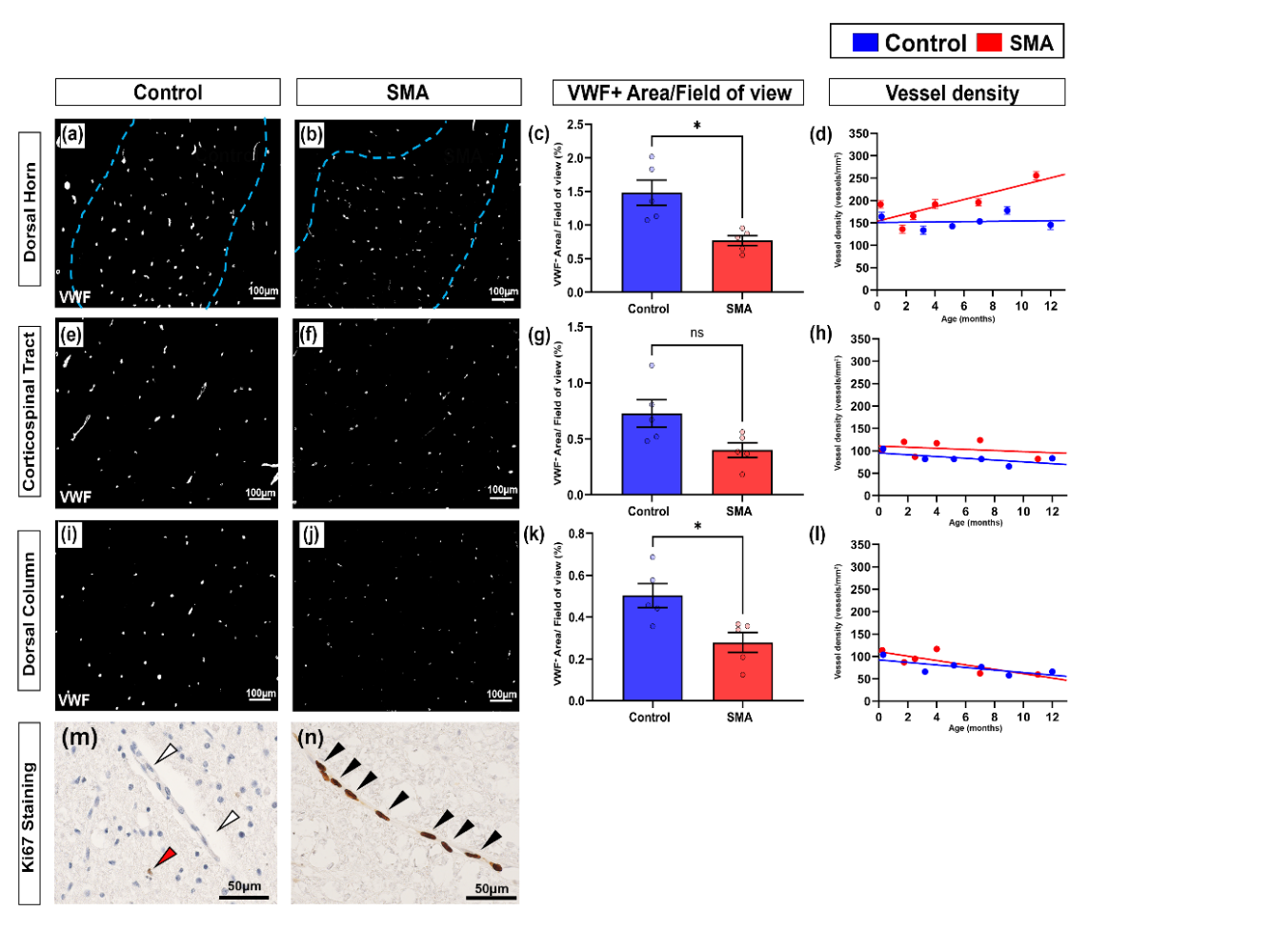


**Supplementary Fig. 1 VWF expression is decreased in multiple regions of the SMA spinal cord**

(a-b) Representative micrographs of (a) control and (b) SMA dorsal horn of the spinal cord labelled with VWF, scale, 100µm. (c) Total VWF^+^ staining per FOV in control and SMA dorsal horn (Welch’s T test, *P <0.05), mean +SEM. (d) Mean vascular density (vessels/mm^2^) in dorsal horn +SEM, with linear regression, 95% CI. (e-f) Representative micrographs of VWF labelled (e) control and (f) SMA corticospinal tract of the spinal cord, scale, 100µm. (g) Total VWF^+^ staining per FOV in control and SMA corticospinal tract (Welch’s T test, where Pns >0.05), mean +SEM. (h) Mean vascular density (vessels/mm^2^) in corticospinal tract +SEM, with linear regression, 95% CI. Control n=6, SMA n=6. (i-j) Representative micrographs of VWF labelled (i) control and (j) SMA dorsal column of the spinal cord, scale, 100µm. (k) Individual case total VWF^+^ staining per FOV in control and SMA dorsal column (Welch’s T test, *P <0.05), mean +SEM. (l) Mean vascular density (vessels/mm^2^) in dorsal column +SEM, with linear regression, 95% CI. For all VWF+ Area/FOV analyses, Control n=5, SMA n=5. For all vascular density analyses, Control n=6, SMA n=6. (m) Representative micrograph of ki67 immunolabelled control spinal cord. White arrowheads show non-proliferative endothelial cells and red arrowhead shows a proliferating cell within the spinal cord parenchyma. (n) Representative micrograph of ki67 immunolabelled SMA spinal cord. Black arrowheads show proliferating endothelial cells.
